# Notes and News

**Published:** 1905-01-21

**Authors:** 


### NOTES AND NEWS.

THE late Mr. Joseph Lambert has bequeathed the ultimate residue of his estate to King Edward's Hospital Fund. This, it is estimated, will produce about £70,000.

LORD STANLEY, Postmaster General, will preside at a banquet in aid of the funds of St. John's Hospital for Diseases of the Skin, Leicester Square, at Claridge's Hotel, on Friday, May 19th.

THE Saxon Snell Prize, in connection with the Royal Sanitary Institute, is now open for competition. The late Mr. Henry Saxon Snell, F.R.I.B.A., left a legacy to found the prize. It is intended to encourage improvements in construction or adaptation of sanitary appliances. It will be awarded by the Council of the Sanitary Institute every three years. The prize consists of £50 and a medal of the Institute. The subject for the competition on the present occasion is "Domestic Sanitary Appliances, with Suggestions for their Improvement." Full particulars of the conditions of the competition can be procured from the Secretary, Royal Sanitary Institute, Margaret Street, W.

LAST week the Princess Christian, in company with Mr. Chamberlain, was present at Liverpool at a lecture delivered before the Liverpool School of Tropical Medicine, by Major Ronald Ross, C.B., F.R.S., on the subject of "The Progress of Tropical Medicine." Sir Alfred Jones presided. Major Ross dwelt upon the impediment in the path of progress which the prevalence of tropical fevers presented. He pointed out that the Liverpool School of Medicine had already sent 14 expeditions to the tropics, and mentioned as the most striking success up to the present date, the achievements at Ismailia, where, owing to sanitary measures being taken the cases of malarial fever fell in one year from 2,000 to 200. Major Ross expressed his belief that the result could be equalled in most fever-stricken districts if the same energy and care were exhibited. He considered the difficulty lay with the local authorities, who could not always be roused to take sufficient interest in the matter.

AT the first meeting of the St. Mary's Hospitals, Manchester, Sir Frank Adam was elected chairman.

THE London County Council have taken to heart the objections urged in THE HOSPITAL, on sanitary grounds, to the carpet covering on the seats of their tramcars on the South London routes; and, having made use of the material they have bought for the purpose, they intend the comfortable wooden seats to be uncovered in future. This is not only economy, but wisdom.

IT is interesting to note the evidences of awakening in the hospital world on the subject of effecting economies. This is the case even in regard to the smaller institutions. For instance, we learn that at the North Devon Infirmary, Barnstaple, the new matron, Miss Lillie, has effected a reduction of expenditure of over £100 on the provisioning of the hospital for the past six months, as compared with the corresponding period of the previous two years. Miss Lillie obtained her housekeeping experience at the East London Hospital for Children, Shadwell, E.

AN interesting case is awaiting judgment at Manchester. The Patent Medicine Vendors' and Drugs Stores Association are suing several well-known chemists for selling ointments marked poison, and admittedly containing poison, to persons unknown to them. The defence is based upon the form in which the poison was sold. Poison, it is argued, is present in many mixtures and articles sold, for example in tobacco, and it would be unreasonable to require the Pharmacy Act to apply in such cases. The Chemist Defence Association is defending, and argues the prosecution has not been brought in the interests of the public.

WE congratulate the committee of Addenbrooke's Hospital, Cambridge, on their appointment of Mr. R. J. Coles as the secretary-superintendent to this hospital, which, though not a large one compared with many in the metropolis and some of the largest provincial medical charities, is distinguished by its position as the clinical centre of one of the most important University Medical Schools. In the smooth and efficient working of such an institution the committee may be greatly assisted by an experienced, energetic, and tactful secretary-superintendent, and, having regard to the ample experience that Mr. Coles has had, firstly at the Warneford Hospital, Leamington, and more recently as secretary and house-governor at the Bristol Royal Infirmary, and the exceedingly good testimonials he received from the presidents of both of these hospitals, we are justified in our expectation that Mr. Coles will continue his successful career at Cambridge to the satisfaction of his new committee and the great advantage of the hospital whose interests will now be his to serve. Mr. Coles is 33 years of age, and he will be missed at Bristol, for unfortunately his wife's health has impelled him to relinquish his work in the West of England, to the regret both of himself and the Committee and medical staff of the Royal Infirmary. There were 292 applicants for the office to which he has been appointed and we cordially congratulate him and wish him every success in his new sphere of work.

